# *trans*-4-[(2,6-Dimethyl­phen­oxy)methyl]cyclo­hexa­necarboxylic acid

**DOI:** 10.1107/S1600536808035502

**Published:** 2008-11-08

**Authors:** Chun-Hong Zhang, Zi-Cheng Li, Hang Song, Wen-Cai Huang

**Affiliations:** a Department of Pharmaceutical and Bioengineering, School of Chemical Engineering, Sichuan University, Chengdu 610065, People’s Republic of China

## Abstract

The title compound, C_16_H_22_O_3_, is a useful inter­mediate in the synthesis of poly(amido­amine) dendrimers. The cyclo­hexane ring adopts a chair conformation. In the crystal structure, mol­ecules are linked into centrosymmetric dimers by pairs of O—H⋯O hydrogen bonds.

## Related literature

For general background on poly(amido­amine) dendrimers, see: Ahmed *et al.* (2001 ▶); Grabchev *et al.* (2003 ▶); Wang *et al.* (2004 ▶). For related structures, see: Bucourt & Hainaut (1965 ▶); Dunitz & Strickler (1966 ▶); Luger *et al.* (1972 ▶).
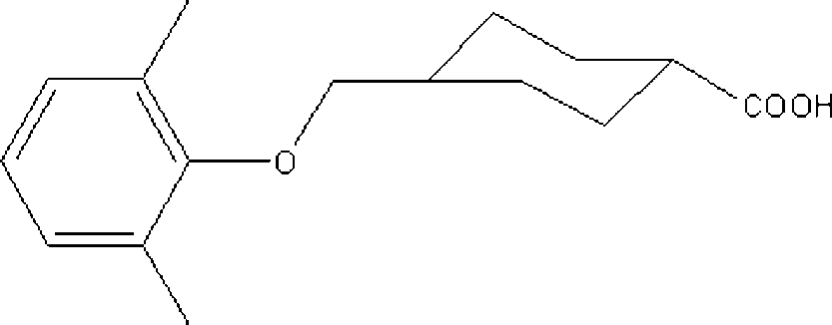


## Experimental

### 

#### Crystal data

C_16_H_22_O_3_
*M* *_r_* = 262.34
Triclinic, 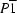

*a* = 7.162 (3) Å
*b* = 7.680 (4) Å
*c* = 14.451 (4) Å
α = 95.26 (4)°
β = 98.35 (4)°
γ = 106.44 (3)°
*V* = 746.9 (6) Å^3^
*Z* = 2
Mo *K*α radiation
μ = 0.08 mm^−1^
*T* = 292 (2) K
0.60 × 0.52 × 0.42 mm

#### Data collection

Enraf–Nonius CAD-4 diffractometer
Absorption correction: none
2886 measured reflections
2715 independent reflections
1461 reflections with *I* > 2σ(*I*)
*R*_int_ = 0.013
3 standard reflections every 250 reflections intensity decay: 2.3%

#### Refinement

*R*[*F* ^2^ > 2σ(*F* ^2^)] = 0.066
*wR*(*F* ^2^) = 0.196
*S* = 1.16
2715 reflections
175 parameters
H-atom parameters constrained
Δρ_max_ = 0.18 e Å^−3^
Δρ_min_ = −0.20 e Å^−3^

### 

Data collection: *DIFRAC* (Gabe & White, 1993 ▶); cell refinement: *DIFRAC*; data reduction: *NRCVAX* (Gabe *et al.*, 1989 ▶); program(s) used to solve structure: *SHELXS97* (Sheldrick, 2008 ▶); program(s) used to refine structure: *SHELXL97* (Sheldrick, 2008 ▶); molecular graphics: *ORTEP-3 for Windows* (Farrugia, 1997 ▶); software used to prepare material for publication: *SHELXL97*.

## Supplementary Material

Crystal structure: contains datablocks global, I. DOI: 10.1107/S1600536808035502/ci2698sup1.cif

Structure factors: contains datablocks I. DOI: 10.1107/S1600536808035502/ci2698Isup2.hkl

Additional supplementary materials:  crystallographic information; 3D view; checkCIF report

## Figures and Tables

**Table 1:** Hydrogen-bond geometry (Å, °)

*D*—H⋯*A*	*D*—H	H⋯*A*	*D*⋯*A*	*D*—H⋯*A*
O2—H2⋯O3^i^	0.82	1.86	2.658 (3)	166

Symmetry code: (i) 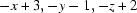.

**Fig. 1.**
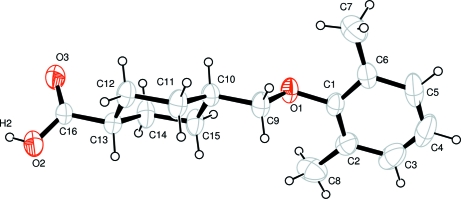
The molecular structure of the title compound. Displacement ellipsoids are drawn at the 50% probability level.

## supplementary crystallographic information

### Comment

Poly(amidoamine) [PAMAM] dendrimers have attracted much interest for their symmetry, high degree of branching and high density of terminal functional groups, and with different structures they could be used in different fields. Various modifications of periphery of PAMAM dendrimers to change its physical or chemical properties have been reported recently (Wang *et al.*, 2004; Grabchev *et al.*, 2003; Ahmed *et al.*, 2001). To change the lipophilicity of PAMAM dendrimers and provide a new type of linker with special stereostructure, a series of cyclohexanic acid derivatives were synthesized. In our synthetic work the title compound was obtained and here we report its crystal structure.

The cyclohexane ring of the title compound (Fig. 1) adopts a chair conformation. The average C—C bond length of the cyclohexane ring is 1.517 (4) Å, which is close to that of *trans*-1,4-cyclohexane dicarboxylic acid (1.523 (3) Å, Luger *et al.*, 1972). The mean endocyclic angle of the cyclohexane is 111.1 (3)°, which is close to that observed for cyclohexane rings (111.1°, Bucourt & Hainaut, 1965; 111.4 (4)°, Dunitz & Strickler, 1966; Luger *et al.*, 1972).

In the crystal structure, the molecules are linked into centrosymmetric dimers by O—H···O hydrogen bonds (Table 1).

### Experimental

Methyl *trans*-4-(tosylmethyl)cyclohexanecarboxylate (3.26 g, 10 mmol), 2,6-dimethylphenol (3.66 g, 30 mmol) and potassium phosphate (10.6 g, 50 mmol) were suspended in dry DMF (20 ml) and heated at 368 K for 8 h, and then water (30 ml) and toluene (30 ml) were added. After agitation, the water layer separated was washed twice with toluene and the organic layer combined was washed with water and then dried with sodium sulfate. After filtration and distillation under vaccum, the crude product obtained was further purified by column chromatography to give pure methyl ester. The ester was then hydrolyzed in a ethanol (15 ml)–1 N NaOH (15 ml) solution for 5 h at 313 K. After cooling and acidification with hydrochloride, the white solid precipitated was collected. Colourless crystals were obtained by slow evaporation in chloroform at room temperature.

### Refinement

H atoms were positioned geometrically (O—H = 0.82 Å and C—H = 0.93–0.98 Å) and refined using a riding model, with *U*_iso_(H) = 1.2–1.5*U*_eq_(C). A rotating group model was used for methyl and hydroxyl groups.

### Crystal data

**Table:**

C_16_H_22_O_3_	*Z* = 2
*M**_r_* = 262.34	*F*_000_ = 284
Triclinic, *P*1	*D*_x_ = 1.167 Mg m^−^^3^
Hall symbol: -P 1	Mo *K*α radiation λ = 0.71073 Å
*a* = 7.162 (3) Å	Cell parameters from 26 reflections
*b* = 7.680 (4) Å	θ = 4.3–7.4º
*c* = 14.451 (4) Å	µ = 0.08 mm^−^^1^
α = 95.26 (4)º	*T* = 292 (2) K
β = 98.35 (4)º	Block, colourless
γ = 106.44 (3)º	0.60 × 0.52 × 0.42 mm
*V* = 746.9 (6) Å^3^	

### Data collection

**Table:**

Enraf–Nonius CAD-4 diffractometer	*R*_int_ = 0.013
Radiation source: fine-focus sealed tube	θ_max_ = 25.5º
Monochromator: graphite	θ_min_ = 1.4º
*T* = 292(2) K	*h* = −8→8
ω/2θ scans	*k* = −5→9
Absorption correction: none	*l* = −17→17
2886 measured reflections	3 standard reflections
2715 independent reflections	every 250 reflections
1461 reflections with *I* > 2σ(*I*)	intensity decay: 2.3%

### Refinement

**Table:**

Refinement on *F*^2^	Secondary atom site location: difference Fourier map
Least-squares matrix: full	Hydrogen site location: inferred from neighbouring sites
*R*[*F*^2^ > 2σ(*F*^2^)] = 0.066	H-atom parameters constrained
*wR*(*F*^2^) = 0.196	*w* = 1/[σ^2^(*F*_o_^2^) + (0.0902*P*)^2^ + 0.0605*P*] where *P* = (*F*_o_^2^ + 2*F*_c_^2^)/3
*S* = 1.16	(Δ/σ)_max_ = 0.001
2715 reflections	Δρ_max_ = 0.18 e Å^−^^3^
175 parameters	Δρ_min_ = −0.20 e Å^−^^3^
Primary atom site location: structure-invariant direct methods	Extinction correction: none

### Special details

**Table:**

Geometry. All e.s.d.'s (except the e.s.d. in the dihedral angle between two l.s. planes) are estimated using the full covariance matrix. The cell e.s.d.'s are taken into account individually in the estimation of e.s.d.'s in distances, angles and torsion angles; correlations between e.s.d.'s in cell parameters are only used when they are defined by crystal symmetry. An approximate (isotropic) treatment of cell e.s.d.'s is used for estimating e.s.d.'s involving l.s. planes.
Refinement. Refinement of *F*^2^ against ALL reflections. The weighted *R*-factor *wR* and goodness of fit *S* are based on *F*^2^, conventional *R*-factors *R* are based on *F*, with *F* set to zero for negative *F*^2^. The threshold expression of *F*^2^ > σ(*F*^2^) is used only for calculating *R*-factors(gt) *etc*. and is not relevant to the choice of reflections for refinement. *R*-factors based on *F*^2^ are statistically about twice as large as those based on *F*, and *R*- factors based on ALL data will be even larger.

### Fractional atomic coordinates and isotropic or equivalent isotropic displacement parameters (Å^2^)

**Table:**

	*x*	*y*	*z*	*U*_iso_*/*U*_eq_	
O1	0.8686 (2)	0.1188 (2)	0.70598 (13)	0.0678 (5)	
O2	1.2419 (3)	−0.5645 (3)	0.96803 (18)	0.0922 (7)	
H2	1.3381	−0.5780	1.0015	0.138*	
O3	1.4736 (3)	−0.3312 (3)	0.93470 (18)	0.0964 (8)	
C1	0.7116 (4)	0.1817 (3)	0.6701 (2)	0.0628 (7)	
C2	0.5908 (4)	0.2148 (4)	0.7308 (2)	0.0769 (8)	
C3	0.4398 (5)	0.2823 (5)	0.6941 (4)	0.1122 (14)	
H3	0.3518	0.3017	0.7324	0.135*	
C4	0.4162 (6)	0.3209 (5)	0.6047 (5)	0.1232 (17)	
H4	0.3148	0.3684	0.5828	0.148*	
C5	0.5403 (6)	0.2907 (4)	0.5465 (3)	0.1052 (13)	
H5	0.5235	0.3191	0.4853	0.126*	
C6	0.6931 (4)	0.2174 (4)	0.5772 (2)	0.0728 (8)	
C7	0.8312 (6)	0.1882 (5)	0.5141 (2)	0.1050 (11)	
H7A	0.9250	0.1368	0.5466	0.157*	
H7B	0.9000	0.3034	0.4965	0.157*	
H7C	0.7576	0.1056	0.4584	0.157*	
C8	0.6272 (6)	0.1818 (5)	0.8314 (3)	0.1143 (13)	
H8A	0.6137	0.0542	0.8333	0.171*	
H8B	0.5326	0.2158	0.8642	0.171*	
H8C	0.7585	0.2541	0.8612	0.171*	
C9	0.8238 (4)	−0.0765 (3)	0.6916 (2)	0.0710 (8)	
H9A	0.8080	−0.1196	0.6249	0.085*	
H9B	0.7005	−0.1320	0.7125	0.085*	
C10	0.9881 (4)	−0.1320 (3)	0.74612 (18)	0.0610 (7)	
H10	1.1123	−0.0654	0.7276	0.073*	
C11	0.9560 (4)	−0.3349 (4)	0.7197 (2)	0.0764 (8)	
H11A	0.9530	−0.3606	0.6524	0.092*	
H11B	0.8287	−0.4032	0.7329	0.092*	
C12	1.1171 (4)	−0.3994 (4)	0.7733 (2)	0.0746 (8)	
H12A	1.2426	−0.3407	0.7551	0.089*	
H12B	1.0871	−0.5308	0.7564	0.089*	
C13	1.1352 (4)	−0.3548 (4)	0.8800 (2)	0.0712 (8)	
H13	1.0096	−0.4209	0.8974	0.085*	
C14	1.1699 (5)	−0.1513 (4)	0.9070 (2)	0.0838 (9)	
H14A	1.2980	−0.0839	0.8942	0.101*	
H14B	1.1718	−0.1259	0.9742	0.101*	
C15	1.0103 (5)	−0.0858 (4)	0.8526 (2)	0.0789 (8)	
H15A	1.0425	0.0460	0.8689	0.095*	
H15B	0.8849	−0.1419	0.8716	0.095*	
C16	1.2952 (4)	−0.4194 (4)	0.9309 (2)	0.0679 (7)	

### Atomic displacement parameters (Å^2^)

**Table:**

	*U*^11^	*U*^22^	*U*^33^	*U*^12^	*U*^13^	*U*^23^
O1	0.0677 (12)	0.0521 (10)	0.0835 (12)	0.0263 (9)	−0.0042 (9)	0.0107 (8)
O2	0.0857 (14)	0.0824 (15)	0.1224 (19)	0.0393 (12)	0.0165 (13)	0.0434 (13)
O3	0.0695 (14)	0.0986 (16)	0.136 (2)	0.0373 (12)	0.0181 (12)	0.0551 (14)
C1	0.0573 (15)	0.0486 (14)	0.0804 (18)	0.0191 (12)	−0.0007 (13)	0.0088 (12)
C2	0.0742 (19)	0.0565 (17)	0.104 (2)	0.0234 (15)	0.0221 (17)	0.0078 (15)
C3	0.083 (3)	0.071 (2)	0.193 (5)	0.0338 (19)	0.041 (3)	0.013 (3)
C4	0.074 (3)	0.079 (3)	0.216 (6)	0.037 (2)	−0.010 (3)	0.029 (3)
C5	0.103 (3)	0.074 (2)	0.122 (3)	0.024 (2)	−0.038 (2)	0.027 (2)
C6	0.0754 (19)	0.0592 (17)	0.0762 (19)	0.0179 (14)	−0.0078 (15)	0.0131 (14)
C7	0.130 (3)	0.105 (3)	0.085 (2)	0.038 (2)	0.024 (2)	0.0224 (19)
C8	0.154 (3)	0.100 (3)	0.102 (3)	0.040 (2)	0.062 (3)	0.012 (2)
C9	0.0734 (18)	0.0548 (16)	0.0841 (19)	0.0268 (13)	−0.0026 (14)	0.0088 (13)
C10	0.0652 (16)	0.0495 (14)	0.0686 (16)	0.0217 (12)	0.0039 (12)	0.0087 (12)
C11	0.0809 (19)	0.0603 (17)	0.090 (2)	0.0345 (14)	−0.0016 (15)	0.0030 (14)
C12	0.0780 (19)	0.0602 (17)	0.090 (2)	0.0348 (14)	0.0035 (15)	0.0051 (14)
C13	0.0676 (17)	0.0716 (18)	0.090 (2)	0.0353 (14)	0.0230 (14)	0.0304 (15)
C14	0.112 (2)	0.081 (2)	0.0727 (19)	0.0588 (19)	0.0024 (16)	0.0080 (15)
C15	0.098 (2)	0.0747 (19)	0.079 (2)	0.0534 (17)	0.0075 (15)	0.0093 (15)
C16	0.0757 (19)	0.0644 (17)	0.0816 (19)	0.0397 (15)	0.0238 (15)	0.0268 (15)

### Geometric parameters (Å, °)

**Table:**

O1—C1	1.397 (3)	C8—H8C	0.96
O1—C9	1.431 (3)	C9—C10	1.505 (3)
O2—C16	1.266 (3)	C9—H9A	0.97
O2—H2	0.82	C9—H9B	0.97
O3—C16	1.256 (3)	C10—C11	1.513 (4)
C1—C2	1.373 (4)	C10—C15	1.522 (4)
C1—C6	1.390 (4)	C10—H10	0.98
C2—C3	1.386 (5)	C11—C12	1.521 (4)
C2—C8	1.496 (5)	C11—H11A	0.97
C3—C4	1.350 (6)	C11—H11B	0.97
C3—H3	0.93	C12—C13	1.526 (4)
C4—C5	1.359 (6)	C12—H12A	0.97
C4—H4	0.93	C12—H12B	0.97
C5—C6	1.402 (5)	C13—C16	1.498 (4)
C5—H5	0.93	C13—C14	1.516 (4)
C6—C7	1.487 (5)	C13—H13	0.98
C7—H7A	0.96	C14—C15	1.521 (4)
C7—H7B	0.96	C14—H14A	0.97
C7—H7C	0.96	C14—H14B	0.97
C8—H8A	0.96	C15—H15A	0.97
C8—H8B	0.96	C15—H15B	0.97
			
C1—O1—C9	113.91 (18)	C9—C10—C15	113.2 (2)
C16—O2—H2	109.5	C11—C10—C15	110.1 (2)
C2—C1—C6	123.7 (3)	C9—C10—H10	107.8
C2—C1—O1	117.7 (3)	C11—C10—H10	107.8
C6—C1—O1	118.5 (3)	C15—C10—H10	107.8
C1—C2—C3	116.5 (3)	C10—C11—C12	112.2 (2)
C1—C2—C8	120.4 (3)	C10—C11—H11A	109.2
C3—C2—C8	123.1 (3)	C12—C11—H11A	109.2
C4—C3—C2	122.2 (4)	C10—C11—H11B	109.2
C4—C3—H3	118.9	C12—C11—H11B	109.2
C2—C3—H3	118.9	H11A—C11—H11B	107.9
C3—C4—C5	120.2 (4)	C11—C12—C13	111.6 (2)
C3—C4—H4	119.9	C11—C12—H12A	109.3
C5—C4—H4	119.9	C13—C12—H12A	109.3
C4—C5—C6	121.2 (4)	C11—C12—H12B	109.3
C4—C5—H5	119.4	C13—C12—H12B	109.3
C6—C5—H5	119.4	H12A—C12—H12B	108.0
C1—C6—C5	116.2 (3)	C16—C13—C14	112.1 (2)
C1—C6—C7	122.8 (3)	C16—C13—C12	110.4 (2)
C5—C6—C7	121.0 (3)	C14—C13—C12	110.0 (2)
C6—C7—H7A	109.5	C16—C13—H13	108.1
C6—C7—H7B	109.5	C14—C13—H13	108.1
H7A—C7—H7B	109.5	C12—C13—H13	108.1
C6—C7—H7C	109.5	C13—C14—C15	111.7 (2)
H7A—C7—H7C	109.5	C13—C14—H14A	109.3
H7B—C7—H7C	109.5	C15—C14—H14A	109.3
C2—C8—H8A	109.5	C13—C14—H14B	109.3
C2—C8—H8B	109.5	C15—C14—H14B	109.3
H8A—C8—H8B	109.5	H14A—C14—H14B	107.9
C2—C8—H8C	109.5	C14—C15—C10	112.6 (2)
H8A—C8—H8C	109.5	C14—C15—H15A	109.1
H8B—C8—H8C	109.5	C10—C15—H15A	109.1
O1—C9—C10	109.9 (2)	C14—C15—H15B	109.1
O1—C9—H9A	109.7	C10—C15—H15B	109.1
C10—C9—H9A	109.7	H15A—C15—H15B	107.8
O1—C9—H9B	109.7	O3—C16—O2	122.8 (2)
C10—C9—H9B	109.7	O3—C16—C13	119.9 (2)
H9A—C9—H9B	108.2	O2—C16—C13	117.3 (3)
C9—C10—C11	109.9 (2)		

### Hydrogen-bond geometry (Å, °)

**Table:**

*D*—H···*A*	*D*—H	H···*A*	*D*···*A*	*D*—H···*A*
O2—H2···O3^i^	0.82	1.86	2.658 (3)	166

Symmetry codes: (i) −*x*+3, −*y*−1, −*z*+2.
